# MalnutritiOn assessment with biOelectrical impedaNce analysis in gastRic cancer patIentS undergoing multimodaltrEatment (MOONRISE)—Study protocol for a single-arm multicenter cross-sectional longitudinal study

**DOI:** 10.1371/journal.pone.0297583

**Published:** 2024-02-06

**Authors:** Zuzanna Pelc, Katarzyna Sędłak, Radosław Mlak, Katarzyna Chawrylak, Katarzyna Mielniczek, Magdalena Leśniewska, Magdalena Skórzewska, Magdalena Kwietniewska, Iwona Paśnik, Katarzyna Gęca, Pieter van der Sluis, Tomasz Banasiewicz, Michał Pędziwiatr, Wojciech P. Polkowski, Timothy M. Pawlik, Teresa Małecka-Massalska, Karol Rawicz-Pruszyński

**Affiliations:** 1 Department of Surgical Oncology, Medical University of Lublin, Lublin, Poland; 2 Department of Human Physiology, Medical University of Lublin, Lublin, Poland; 3 Department of Clinical Pathomorphology, Medical University of Lublin, Lublin, Poland; 4 Upper Gastrointestinal Department of Surgery, Erasmus MC, Rotterdam, The Netherlands; 5 Department of General, Endocrynological Surgery and Gastrointestinal Oncology, Institute of Surgery, Poznan University of Medical Sciences, Poznań, Polska; 6 2nd Department of Surgery, Jagiellonian University Medical College, Kraków, Polska; 7 Department of Surgery, The Ohio State University Wexner Medical Center and James Cancer Center, Columbus, Ohio, United States of America; Ehime University Graduate School of Medicine, JAPAN

## Abstract

European data suggests that over 30% of gastric cancer (GC) patients are diagnosed with sarcopenia before surgery, while unintentional weight loss occurs in approximately 30% of patients following gastrectomy. Preoperative sarcopenia significantly increases the risk of major postoperative complications, and preoperative body weight loss remains a superior predictor of outcome and an independent prognostic factor for overall survival (OS) in patients with GC. A standardized approach of nutritional risk screening of GC patients is yet to be established. Therefore, the MOONRISE study aims to prospectively analyze the changes in nutritional status and body composition at each stage of multimodal treatment among GC patients from five Western expert centers. Specifically, we seek to assess the association between nutritional status and body composition on tumor response following neoadjuvant chemotherapy (NAC). Secondary outcomes of the study are treatment toxicity, postoperative complications, quality of life (QoL), and OS. Patients with locally advanced gastric adenocarcinoma scheduled for multimodal treatment will be included in the study. Four consecutive nutritional status assessments will be performed throughout the treatment. The following study was registered in ClinicalTrials.gov (Identifier: NCT05723718) and will be conducted in accordance with the STROBE statement. The anticipated duration of the study is 12–24 months, depending on the recruitment status. Results of this study will reveal whether nutritional status and body composition assessment based on BIA will become a validated and objective tool to support clinical decisions in GC patients undergoing multimodal treatment.

## Introduction

Gastric cancer (GC) remains the fifth most diagnosed cancer and the fourth leading cause of cancer-related death worldwide [1]. By 2040, the annual burden of GC is expected to occur with around 1.8 million new cases and 1.3 million deaths [2]. While the introduction of perioperative chemotherapy and advancement in surgical quality improved overall survival (OS) [3], there is a constant need to focus on prevention and individualized therapy to further reduce morbidity and mortality of GC patients [4–6].

In Europe, multimodal therapy consisting of neoadjuvant chemotherapy (NAC), gastrectomy with adequate lymph node (LN) dissection, followed by adjuvant chemotherapy (AC), is the gold standard of care, particularly in locally advanced stages [7]. Following the results of the FLOT4-AIO trial, which compared fluorouracil plus leucovorin, oxaliplatin, and docetaxel (FLOT regimen) versus fluorouracil or capecitabine plus cisplatin and epirubicin (ECF/ECX regimen) for locally advanced, resectable gastric or gastro-oesophageal junction adenocarcinoma, perioperative FLOT chemotherapy has been established as a standard of care [8]. Given its notable toxicity rate, it is advised for a medically fit group of patients. Of note, the assesment of physical fitness as well the correlation between FLOT and nutritional status have not beed comprehensively investigated.

Malnutrition contributes to increased morbidity, higher risk of perioperative complications, and systemic toxicity ultimately leading to prolonged hospital stays and diminished quality of life (QoL) [9]. European data suggests that over 30% of GC patients are diagnosed with sarcopenia before surgery [10], whereas in East the incidence of preoperative malnutrition in geriatric population is estimated to reach up to 65% [11]. While unintentional weight loss occurs in approximately 30% of patients following surgery [12], Decresed Free Fat Mass (FFM) is reported to be associated not only with worse OS but also with higher rates of postoperative complication in GC patients undergoing gastrectomy [13]. Additionally, the curative-intent surgery introduces more significant changes in nutritional status, elevating the risk of post-operative complications compared with palliative management. Conversely, visceral and sarcopenic obesity are associated with cancer progresssion and cancer-related comorbidities. Notably, preoperative sarcopenia significantly increases the risk of major postoperative complications (OR 1.67, 95%CI 1.14–2.46) [14], and preoperative body weight loss remains a superior predictor of outcome and an independent prognostic factor for OS in patients with GC [4]. Additionally, malnutrition implicates tumor progression and a more aggressive disease biology associated with several malignancies, including GC [15].

Tumor regression grade (TRG) is the percentage of viable cancer cells in the resected primary tumor, which defines histopathological response of neoadjuvant chemotherapy [16]. In addition to strong correlation with Lauren’s classification, TNM stage, and tumor grading, TRG is an important prognostic factor of OS for locally advanced GC patients undergoing multimodal treatment, as demonstrated in a recent large single-center cohort from Germany [17]. Of note, TRG is a better prognostic factor than the Response Evaluation Criteria in Solid Tumors (RECIST)–the most widely used tumor response classification [18]. In an Italian retrospective study, authors assessed the relation between immune-nutritional status and TRG among resectable GC patients [19]. Naples Prognostic Scale (NPS) was implemented to verify patient nutritional condition. Multivariate analysis revealed that TRG was an independent prognostic variable and NPS evaluation enables prediction of the benefit from NAC. Therefore, improving immune-nutritional conditions may influence tumor downstaging.

To date, variable tools have been implemented for the screening of malnutrition: Nutritional Risk Screening (NRS), Subjective Global Assessment (SGA), serum albumin or BMI. Limitations of these methods result from dependence on confounding variables, including not validated laboratory norms and tools for specific patient populations. Park et al. developed a prediction model for screening GC patients at risk of malnutrition undergoing surgery [20]. The predictive variables were age, sex, preoperative BMI and malnutrition status, cancer stage, operation method and type of reconstruction, chemotherapy, and presence of postoperative complications. A significant decrease in BMI was observed, reaching a severe level in over 20% of patients. Independent risk factors associated with malnutrition included the type of surgery (total and proximal), low preoperative BMI and female sex. Nonetheless, due to the considerable heterogeneity of nutritional status assessment, the most adequate method to evaluate the nutritional status of GC patients undergoing multimodal treatment has yet to be established.

An objective and non-invasive technique to distinguish changes in body composition over time is bioelectrical impedance analysis (BIA) [21]. These measurements are possible due to body component resistance, reactance, and recording a voltage drop in the applied current. Resistance is the restriction to the flow of an electric current, dependent on the amount of water present in tissues. Reactance results from the charge produced by the cell membranes. In the systemiatic review and meta-analysis of 8402 GC patients, only 9 out of 38 studies implemented BIA in nutritional status assessment, with a single report from Europe [14]. Although BIA requires standardization, it remains an objective, repeatable, safe and cost efficient tool for body composition analysis [21]. Moreover, BIA reveals the primary signs of malnutrition even several months before cachexia [22].

Although previous studies highlighted the impact of nutritional status on the outcomes of antitumor therapy [23], body composition has been observed to correlate with the response to preoperative chemotherapy among patients breast and lung cancer patients [24]. Therefore, the MOONRISE study aims to prospectively analyze the changes in nutritional status and body composition at each stage of multimodal treatment among GC patients from five Western expert centers. Specifically, we seek to assess the association between nutritional status and body composition on tumor response following NAC. Secondary outcomes of the study are treatment toxicity, postoperative complications, QoL, and OS.

## Materials and methods

### 1. Study design

This prospective single-arm multicenter cross-sectional longitudinal study was registered in ClinicalTrials.gov (Identifier: NCT05723718) and approved by Medical University of Lublin Bioethical Committee Board (Ethic Code: KE-0254/245/12/2022). The research includes patients with histologically confirmed, potentially curative gastric adenocarcinoma scheduled for multimodal treatment by the decision of multidisciplinary tumor board. Data will be collected from a prospectively maintained database of all GC patients scheduled for multimodal treatment. Written informed consent will be obtained from each patient prior to study inclusion. The participant’s consent will be obtained prior to the beginning of the study, during the hospital stay for staging laparoscopy. The surgeon will gain consent after thoroughly explaining the study’s schedule and objectives. All procedures will be performed in accordance with the current revisions of the Declaration of Helsinki. The following study will be conducted in accordance to the Strengthening of Reporting of Observational Studies in Epidemiology (STROBE) statement. The anticipated duration of the study is 12–24 months, depending on the recruitment status. Any protocol modifications including changes to eligibility criteria, outcomes, analyses will be updated on ClinicalTrials.gov.

### 2. Study population

Patients will be assessed for eligibility to participate in this study after verifying the following criteria and signing informed consent.

#### 2.1 Inclusion criteria

1. Age ≥ 18 years

2. Histologically confirmed gastric adenocarcinoma (or undifferentiated carcinoma)

3. Stage II–III disease (cT2 cN+ and cT3-T4 cN+/-) based on the 8th edition of TNM classification [25, 26].

4. Qualification for multimodal treatment by the decision of the multidisciplinary tumor board

#### 2.2 Exclusion criteria

1. Early GC (cT1N0-3M0) scheduled for endoscopic treatment by multidisciplinary team

2. Gastric stump carcinoma

3. Distant metastasis

4. Upfront surgery

5. Other malignancies

6. Contraindications to BIA (e.g., implanted cardiac devices, metal implants or pregnancy)

#### 2.3 Definition of malnutrition

The definition of malnutrition is based on the ESPEN Malnutrition Diagnostic Criteria [27] as BMI < 18.5 kg/m^2^. The ESPEN Consensus specifies that before a diagnosis of malnutrition can be made, the patient must meet "nutritional risk" criteria according to any validated nutritional risk screening tool [28, 29]. The diagnosis is then confirmed by fulfilling either of two alternative sets of diagnostic criteria: BMI <18.5 kg/m^2^, according to the World Health Organization (WHO) definition of underweight, or a concurrent finding of weight loss along with reduced BMI (age-dependent cutoff values) or reduced fat-free mass index (gender-dependent cutoff values) [30]. ESPEN defines cancer cachexia as a specific form of chronic disease-related malnutrition with inflammation defined as loss of >5% body weight or weight loss >2% together with BMI decrease <20 kg/m^2^ or reduction of fat-free mass [31], i.e. appendicular skeletal muscle mass index <7.2 kg/m^2^ in men or <5.5 kg/m^2^ in women accompanied by elevated serum C-reactive protein (CRP) concentration and/or decreased serum albumin concentration [27, 32]. For this study, the nutritional status will be assessed parallelly using both ESPEN definitions.

### 3. Study procedures

#### 3.1 Nutritional status assessment

Nutritional status assessment will include:

1. Selected clinical variables evaluation (including weight, BMI, nutritional status scales)

2. BIA (including FFM)

3. Selected laboratory parameter assessment (including albumin level, CRP)

After inclusion in the study, each patient will have four consecutive nutritional status assessments:

1. One day prior SL or during qualification to NAC (maximum two weeks before the start of treatment).

2. One day before the gastrectomy.

3. One month after the gastrectomy.

4. After the last cycle of AC.

#### 3.2 Clinical variables evaluation

The primary demographic and clinical data of each patient (e.g., age, gender, comorbidities, body measurements, details of histopathological reports, Lauren classification), results of clinical evaluation, including diagnostic imaging methods, performance status and nutritional status scales assessment will be collected by taking a medical history, performing a physical examination, and reviewing patient records. All data will be confidential and stored in an anonymous database.

During each hospital stay, patients will be assessed for:

- Performance status following the Eastern Cooperative Oncology Group (ECOG)/WHO scale
- Muscle strength (dynamometer)
- SGA scale, NRS scale, Short Form Dietary Questionnaire (SFDQ)
- Nutritional Risk Index (NRI)
- BMI
- QoL based on gastric cancer-specific health-related quality of life questionnaire (EORTC QLQ–STO22)

*3*.*2*.*1 BIA*. After inclusion in the study, each patient will have four BIA assessments:

1. One day prior SL or during qualification to NAC (maximum two weeks before the start of treatment).

2. One day before the gastrectomy.

3. One month after the gastrectomy.

4. After the last cycle of AC.

BIA will include directly measured parameters:

- electric properties of body’s tissues: membrane capacitance, resistance, impedance, reactance, phase angle
- calculated parameters reflecting body composition: extracellular water (ECW), intracellular water (ICW), total body water (TBW), FFM, fat mass, Body Composition Monitor (BCM), extracellular mass (ECM), ECM/BCM ratio, muscle mass

*3*.*2*.*2 Laboratory parameters assessment*. Nutritional status assessment results will be correlated with standard laboratory tests performed concurrently with such assessment as well as before each cycle of chemotherapy, including:

- morphology,
- biochemistry (e.g., creatinine, uric acid, liver tests, serum albumin, total protein test, triglyceride, total cholesterol, low-density lipoprotein (LDL), high-density lipoprotein (HDL) level)
  lipid profile will only be performed along with the nutritional assessments
- electrolytes (e.g., sodium, potassium, magnesium)
- immunohistochemistry (Ca 72–4, Ca19-9, CEA)
- inflammatory markers (CRP and IL- 6 level)

Depending on individual oncologist decision, evaluation will include:

- pregnancy test (at the baseline)
- iron, ferritin, transferrin

#### 3.3 Multimodal treatment

*3*.*3*.*1 Perioperative chemotherapy*. The first choice of perioperative chemotherapy will be based on the Fluorouracil, Leucovorin, Oxaliplatin, and Docetaxel (FLOT) protocol, administered four cycles before and four cycles after the gastrectomy every two weeks. The regimen follows National Comprehensive Cancer Network (NCCN) guidelines [33]: docetaxel at 50 mg/ml, oxaliplatin at 85 mg/ml, leucovorin at 200 mg/ml and fluorouracil at 2600 mg/ml. A gastrectomy will be scheduled for at least four weeks after the last dose of NAC. In case of contraindication to docetaxel, the patients will be scheduled for FOLFOX (oxaliplatin 85 mg/m2, leucovorin 200 mg/m2, 5-FU bolus 400 mg/m2 and then 5-FU 2,400 mg/m2 as a continuous infusion over 46 h repeated every 2 weeks) or FLO (oxaliplatin at 85 mg/ml, leucovorin at 200 mg/ml and fluorouracil at 2600 mg/ml over 24 h each 2 weeks) regimen. Before qualification for the treatment is confirmed an assessment will be performed including computed tomography (CT) scan, staging laparoscopy with peritoneal lavage, and cytological evaluation.

*3*.*3*.*2 Gastrectomy*. After obtaining written consent, patients will be scheduled for surgery performed by an experienced surgeon with adequate LN dissection based on tumor pathology, size, and location. The following perioperative surgical data will be registered:

- Type of surgery (open / laparoscopic / robotic)
- Extent of gastrectomy (total / proximal / distal)
- Method of reconstruction (Billroth I / Billroth II / Roux-en-Y / Double-Tract)
- Extent of lymphadenectomy (D1 / D1+ / D2 / D2+ / D3)
- Operative time
- Blood loss
- Comprehensive Complication Index
- Textbook Oncological Outcome (TOO) [34]

Additionally, a following histopathological data will be registered:

- Lauren histological type
- (y)pTNM
- Grading
- Radicality of resection
- TRG according to Becker’s system

*3*.*3*.*3 Assessment of the response to NAC*. Tumor response to NAC will be evaluated according to the histopathologic regression based on Becker’s TRG system [35, 36]. This scale categorizes regression of the primary tumor into a 4-stage grading system:

- Grade 1—complete response (no residual tumor)
- Grade 2—subtotal regression (<10% residual tumor)
- Grade 3—partial regression (10–50% residual tumor)
- Grade 4—no regression (>50% residual tumor)

All patients will be divided into two cohorts according to the TRG: NAC responders (TRG = 1, 2) and non-responders (TRG = 3, 4).

#### 3.4 Follow-up

Follow-up will be conducted for five years after the end of the treatment. All patients will be monitored in an outpatient clinic every three months for the first year, then every six months up to the third year after finishing the treatment and subsequently every 12 months up to 5 years after the end of active treatment [36, 37].

The follow-up will consist of:

- physical examination (weight, BMI, nutritional status, performance status) hospital admission details during follow-up (date admission and discharge, hospital, indication, relation to study treatment and intervention)
- QoL based on EORTC QLQ–STO22 questionnaire
- treatment-related toxicity and complications
- blood tests (e.g., tumor markers, morphology, biochemistry, inflammatory markers)
- monitoring the nutritional defincies (B12, iron, calcium, NRI)
- CT scan of the abdomen–first one in the third month after finishing the treatment, then every 6 months up to 3 years and once a year until the fifth year after the end of the treatment (unless the oncologist decides otherwise due to symptoms and laboratory or clinical manifestations)
- survival status (death/ alive, date of death, cause of death)

#### 3.5 Schedule of the study

**Fig 1:**
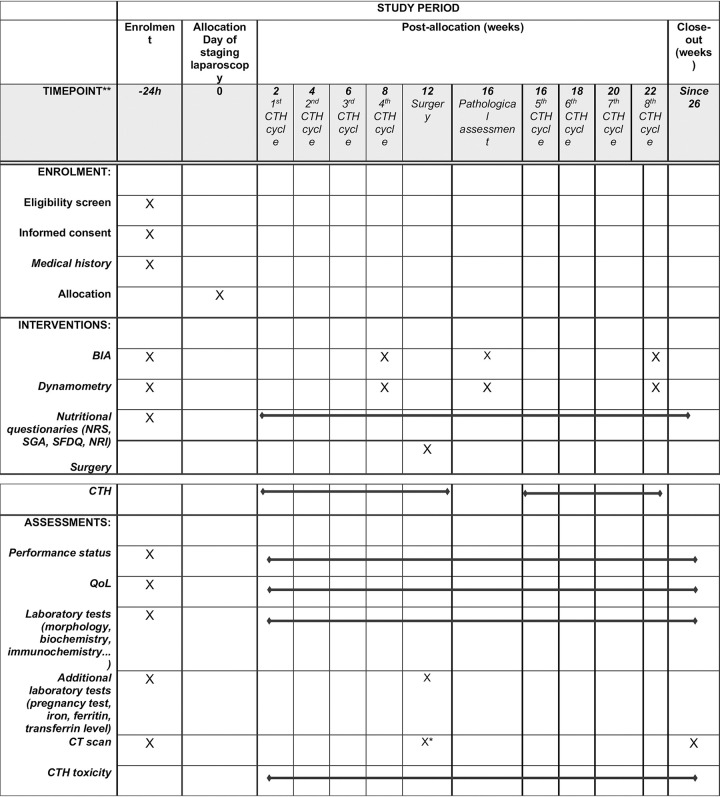
SPIRIT schedule for MOONRISE study. QoL—quality of life, BIA—bioimpedance analysis, NRS—nutrition risk screening, SGA—Subjective Global Assessment, SFDQ—Short Form Dietary Questionnaire, NRI—nutritional risk index, CT—computed tomography, CTH—chemotherapy, *—non-obligatory, according to the decision of the oncologist.

A detailed schedule of the study is presented in Fig 1.

**Fig 2:**
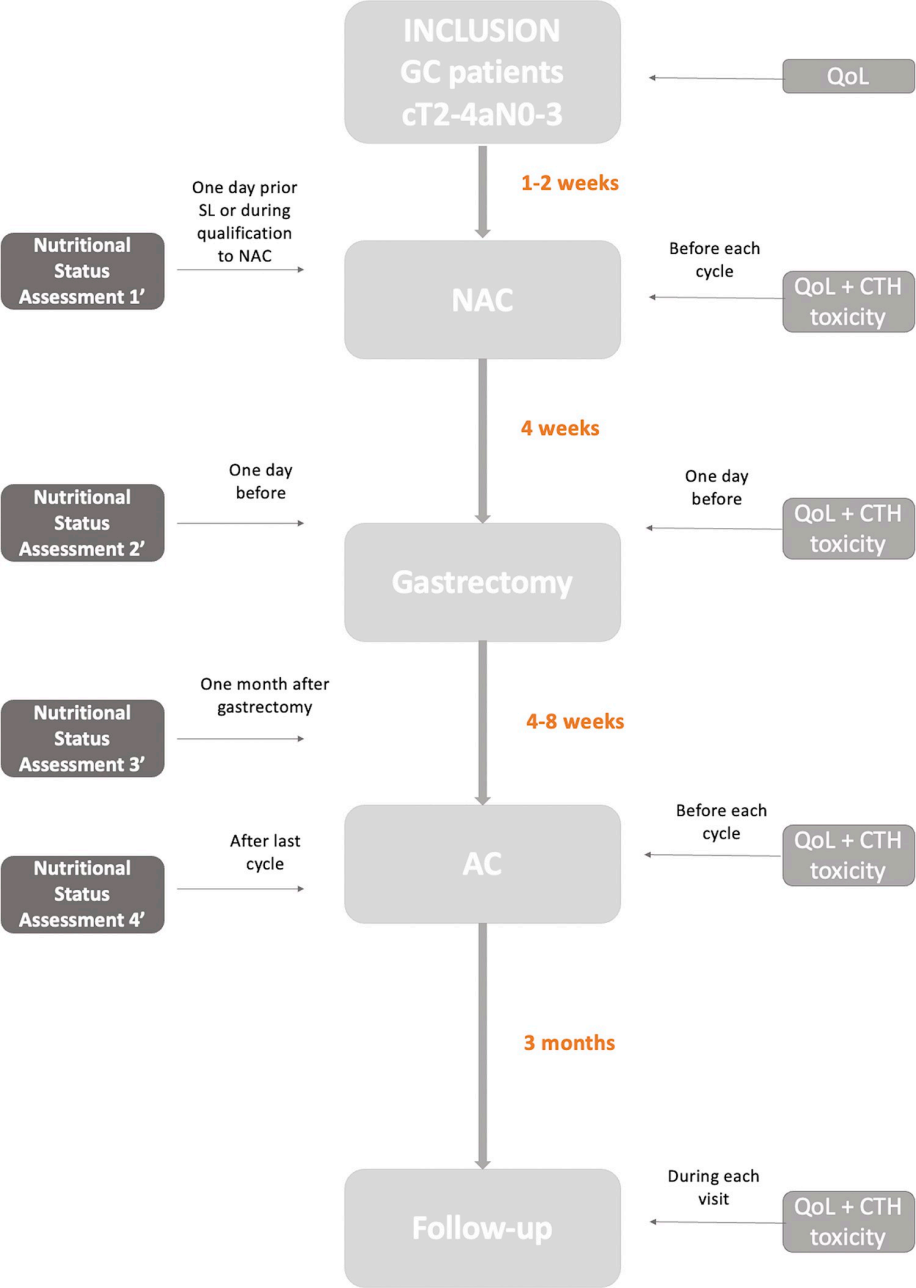
Flowchart of the study.

An algorithm of the clinical pathway and the time points for nutritional status assessment are shown in Fig 2.

GC–gastric cancer, NAC–neoadjuvant chemotherapy, QoL–quality of life, CTH–chemotherapy, AC–adjuvant chemotherapy

**Fig 3:**
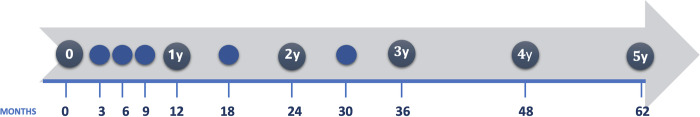
Schedule of the follow-up visits.

The frequency of follow-up visits is depicted in Fig 3.

#### 3.6 Statistical analysis

MedCalc v.15.8 (MedCalc Software, Belgium) will be used for statistical data analysis. D’Agostino-Pearson will be used to assess the normality of the data distribution. Depending on the continuous data distribution type, the mean and standard deviation or median and interquartile range / minimum-maximum range will be used as a measure of data concentration and spread (for normally and non-normally distributed data, respectively). Moreover, depending on the continuous data distribution type, parametric (t-test, Pearson’s correlation) or non-parametric tests (e.g., U-Mann-Whitney, Wilcoxon, Spearman’s correlation) will be used (for normally and non-normally distributed data, respectively). Categorized and dichotomized variables will be expressed as numbers and percentages. Chi-square or Fisher exact test will be used to assess the statistical difference in data distribution according to the studied groups. The test odds ratio (OR) and corresponding 95% confidence intervals (CI) will be used to assess the chance/risk of an occurrence of a particular phenomenon. Logistic regression models will be used in the multivariable analysis to assess chance/risk of an event of a specific phenomenon. OS will be defined as the time from the date of surgery to the date of patient death or the date of the last follow-up. The log-rank test will be used to calculate the proportional hazard ratio and the corresponding 95% CI in univariable OS analysis (the Kaplan-Meier estimation method will be used to generate survival curves), whereas Cox logistic regression models will be used in multivariable OS analysis. In all analyses, a two-sided p-tests will be used, and results with a p-value below 0.05 will be considered statistically significant.

#### 3.7 Sample size calculation

Due to the lack of studies evaluating the value of malnutrition on TRG in patients with advanced GC undergoing multimodal treatment, we decided to calculate sample size based on data retrieved in our facility, which resulted from comparison of percentages of patients with and without malnutrition defined exactly according to ESPEN recommendations (since the FFM assessment method was not indicated, taking into consideration its advantages we decided to use for this purpose BIA measurements) and primary endpoint–response to NAC according to TRG (TRG1 or TRG2—responders vs TRG3 or TRG4—non-responders). Most medical studies consider a p-value below 0.05 to reject the null hypothesis, thus type I error (alpha) of 0.05 value was used. In the case of type II error, we set a cut-off of beta on 0.2 to achieve 80% of statistical power. Considering the percentage of patients without (55%) and with (83.3%) malnutrition that have not responded to NAC as well as the ratio of sample sizes in compared groups (2.2:1), the minimal study group was estimated as 125 patients.

#### 3.8 Quality assurance

The quality assurance team associated with this study will include clinical oncology, oncological surgery, pathology, and radiology experts. Data censors will communicate with branch centers and randomly check the quality of data collection.

#### 3.9 Data collection and management

Each center will have at least two physicians to enroll patients in this study and arrange therapy during multidisciplinary team meetings. Two physicians will collect and secure data at their centers. All electronic documents will be confidential. The database will be under the project leader’s supervision, and no researcher will be allowed to use the data unless permitted.

## Discussion

Despite the implementation of multimodal treatment for GC patients in the West for over a decade, overall relative survival rate remains low [38]. Introduction of minimally invasive gastrectomy in Europe did not improve short-term outcomes and early survival, as demonstrated in the recent results of LOGICA trial [39]. Moreover, an ancillary study of this trial demonstrated that preoperative low skeletal muscle mass and high visceral and subcutaneous adipose tissue radiation attenuation (indicating fat depleted of triglycerides) were associated with a higher risk of developing major postoperative complications among patients undergoing multimodal treatment [40]. Noteworthy, body composition was assessed with CT image analysis, which is inconsistent with BIA. Therefore, the prognostic value of nutritional status and body composition in GC patients undergoing multimodal treatment remains unclear [14].

Malnutrition may not only cause postoperative complications, but also reduce the rate of surgical radicality in patients undergoing cytoreductive surgery and hyperthermic intraperitoneal chemotherapy (HIPEC) [41], a subpopulation of particularly high risk of recurrence and low survival. Since GC patients with acceptable QoL after perioperative chemotherapy and radical surgery have improved survival, as recently shown in CRITICS trial [42], the secondary outcomes of this study will include chemotherapy toxicity, postoperative complications and QoL assessment.

The role of malnutrition in GC patients has been evaluated extensively in the East, where upfront surgery remains the most common treatment option [11, 20, 21, 43]. In a large retrospective analysis by Zheng et al., among 1976 patients included in the study cohort, 412 (21%) were malnourished [44]. The overall incidence of complications in the malnourished group was significantly higher than in the well-nourished group (21.4 vs. 15.5%). Moreover, the malnourished group had significantly lower OS and disease free-survival (DFS) - 59.1 vs. 75% and 54.8 vs. 72.5%, respectively. Nonetheless, these results cannot be directly extrapolated into the Western population due to diverse racial constituents and different recommendations for managing locally advanced GC cases [45].

Data from the U.S. suggest that malnourished patients undergoing gastrectomy are at higher risk of wound complications, respiratory failure, and death [43]. Although the need for early identification of nutritional deficiencies has been highlighted, most studies evaluating the clinical impact of malnutrition in GC patients were based on single, non-objective parameters that, in contrast to BIA, do not reflect actual body composition [20, 46, 47].

To date, no published research on GC patients treated with multimodal therapy has used the same recommended definition of malnutrition, which probably had led to a significant underestimation of its occurrence and biased previous results. The Italian cohort study was the first to assess the correlation between GC patients undergoing FLOT chemotherapy and body composition changes based on retrospective CT scan analysis [48]. The preliminary results showed a significant influence between systemic therapy and sarcopenia, a decrease in BMI, and visceral adipose. TRG was associated with skeletal muscle index. However, these findings did not influence short-term treatment outcomes such as completion of preoperative FLOT or qualification for gastrectomy. The present research applies both ESPEN diagnostic criteria for malnutrition and cachexia based on BMI and FFM. These parameters can be objectively and repetitively evaluated by BIA.

The current study has several limitations. GC represents substantial molecular and phenotypical heterogeneity [49], which may affect the study outcomes. However, multicenter collaboration, histological evaluation, strict inclusion and exclusion criteria and representative sample size to increase the statistical power of the analysis, should collectively mitigate the risk of potential bias. Furthermore, the results obtained by BIA may be influenced by the patient’s hydration status, unusual body structure, or extreme BMI. Therefore, BIA-derived parameters, such as Bioelectrical Impedance Vector Analysis (BIVA) and Phase Angle (PhA), could be utilized. BIVA assesses hydration status and cell mass independently of body weight and height [50]. Furthermore, by converting BIVA measurements to z-scores, body composition across various variables such as gender, cancer histology type, and disease stage can be compared. Additionally, multivariable analysis will be performed, to address all potentially confounding factors.

## Conclusions

While the FLOT regimen has positively impacted long-term outcomes, with estimated 5-year OS rates of 40%, there is a need for enhanced treatment strategies to further improve survival and QoL in GC patients. Investigating the association between nutritional status and pathologic response assessed by TRG presents an opportunity to tailor treatment decisions in comprehensive cancer care. However, the lack of standardized nutritional assessment in GC patients leads to underdiagnosis of nutritional impairment. Additionally, specific exercise and dietary recommendations for GC prehabilitation has not been well-established yet. The MOONRISE study aims to address these clinical gaps by utilizing BIA-derived measures, showing promising potential in preoperative risk evaluation, and reducing postoperative complications. Results of the proposed study will reveal whether nutritional status and body composition assessment based on BIA will become an objective tool to support multimodal treatment of GC patients.

## Supporting information

S1 File Translated resolution of bioethical committee of Medical University of Lublin and study protocol in English. (DOCX)

S2 File Study protocol in Polish. (DOCX)
