# Supplementary material for: MalnutritiOn assessment with biOelectrical impedaNce analysis in gastRic cancer patIentS undergoing multimodaltrEatment (MOONRISE)—Study protocol for a single-arm multicenter cross-sectional longitudinal study
Source: PLoS One. 2024 Feb 6;19(2):e0297583. doi: 10.1371/journal.pone.0297583 (PMC10846730; doi:10.1371/journal.pone.0297583)
Supplement: S1 File — (DOCX) [file pone.0297583.s001.docx]

**Resolution of Bioethical Committee of Medical University of Lublin**

**No KE-0254/245/12/2022**

On 15th December 2022, the Bioethical Committee of the Medical University of Lublin became acquainted with the study protocol:

*“Prospective, multicenter study evaluating the nutritional status with bioelectrical impedance analysis in gastric cancer patients undergoing multimodal treatment (MOONRISE study).”*

RESEARCH THEME LEADER:

Zuzanna Pelc, MD

Department of Surgical Oncology

Medical University of Lublin

Radziwiłłowska 13 street

20-080 Lublin

The Committee received the following documents:

1. Request to the Bioethical Committee
2. Study Protocol
3. Informed consent form
4. Information for survey participants
5. Consent to processing of personal data
6. Model declaration of acceptance of liability insurance by the participant
7. Model questionnaire
8. Approval of the Head of the Department to conduct the study
9. Request for an opinion fee waiver

After evaluating the study protocol under the Guidelines for Good Clinical Practice (GCP) regulations, the Bioethical Committee **approved** the study protocol.

This opinion expires upon completion of the study.

The approval was signed by

**Head of the Committee – prof. Jolanta Szymańska**

Members of the Committee:

| No. | Name and last name | Profession | Signature |
| --- | --- | --- | --- |
| 1. | Prof. Jolanta Szymańska, | Dentist |  |
| 2. | Hanna Czekajska-Łuckiewicz, PhD | Pharmacist |  |
| 3. | Prof. Iwona Beń-Skowronek | Pediatrician |  |
| 4. | Prof. Hanna Karakuła-Juchnowicz, | Psychiatrist |  |
| 5. | Prof. Elżbieta Czekajska-Chehab | Radiologist |  |
| 6. | Prof. Witold Zgodziński | Surgeon |  |
| 7. | Prof. Paweł Krawczyk | Internist |  |
| 8. | Prof. Wojciech Polkowski | Surgical oncologist |  |
| 9. | Prof Janusz Kocki | Geneticist |  |
| 10. | Prof. Grzegorz Dzida | Internist, diabetologist |  |
| 11. | Krzysztof Smykowski, PhD | Preist |  |
| 12. | Lidia Sierpińska, PhD | Nurse |  |
| 13. | Stanisław Pęzioł | Solicitor |  |

**STUDY PROTOCOL**

**STUDY TITLE:**

**Prospective, multicenter study evaluating the nutritional status with bioelectrical impedance analysis in gastric cancer patients undergoing multimodal treatment (MOONRISE study).**

**RESEARCH THEME LEADER:**

Zuzanna Pelc, MD, PhD

**INSTITUTION:**

Medical University of Lublin

Department of Surgical Oncology

**TELEPHONE NUMBERS RELATING TO THE STUDY:**

Department of Surgical Oncology, Medical University of Lublin

81-531-81-26

Gastric cancer (GC) remains one of the most commonly diagnosed malignancies and the fourth leading cause of oncological death worldwide. Using perioperative chemotherapy and advances in surgical quality have improved treatment outcomes. However, there is a continued need to individualize therapy to reduce morbidity and mortality in oncology patients.

Despite advances in the understanding of GC biology, surgical treatment remains the standard of care for locally advanced disease. The introduction of multimodal treatment into clinical practice requires verification of the optimal extent of excision of both the primary tumor and regional lymph nodes.

The use of neoadjuvant chemotherapy increases the radicality of resection and eliminates early disease dissemination while allowing clinical and histopathological assessment of response to treatment.

Although preoperative treatment increases the resectability rate of the primary tumor, its toxicity reduces patients' quality of life. In addition, approximately 30% of GC patients experience unintentional weight loss after surgical treatment, and in patients over 65 years of age, the malnutrition rate reaches up to 70%.

Malnutrition contributes to increased morbidity, higher rates of perioperative complications, and systemic toxicity. It results in prolonged hospital stay, reduced quality of life, and decreased survival. The correlation between nutritional status and overall survival in patients with locally advanced GC treated with multimodal therapy is inconclusive. However, independent prognostic factors are weight loss, both preoperatively and postoperatively. Furthermore, malnutrition promotes the progression and aggressive course of the disease. Chronic inflammation impairs the immune system and increases the risk of postoperative infection and the spread of the disease.

# One non-invasive and objective method of tracking changes in body composition is bioelectrical impedance analysis (BIA). BIA involves measuring impedance (a type of electrical resistance made up of resistance and reactance of tissues through which a low current - 0.8-1mA - is passed). Resistance is the restriction to the flow of an electric current, while reactance results from the electrical capacitance and structure of cell membranes. BIA can reveal primary signs of malnutrition even months before cachexia, and the lack of radiation exposure and low cost of the test allows changes in body composition to be tracked regularly.

# Study design

This prospective, multicenter observational study aims to assess the nutritional status using BIA in patients with advanced GC scheduled for multimodal therapy.

**Flowchart of the study**


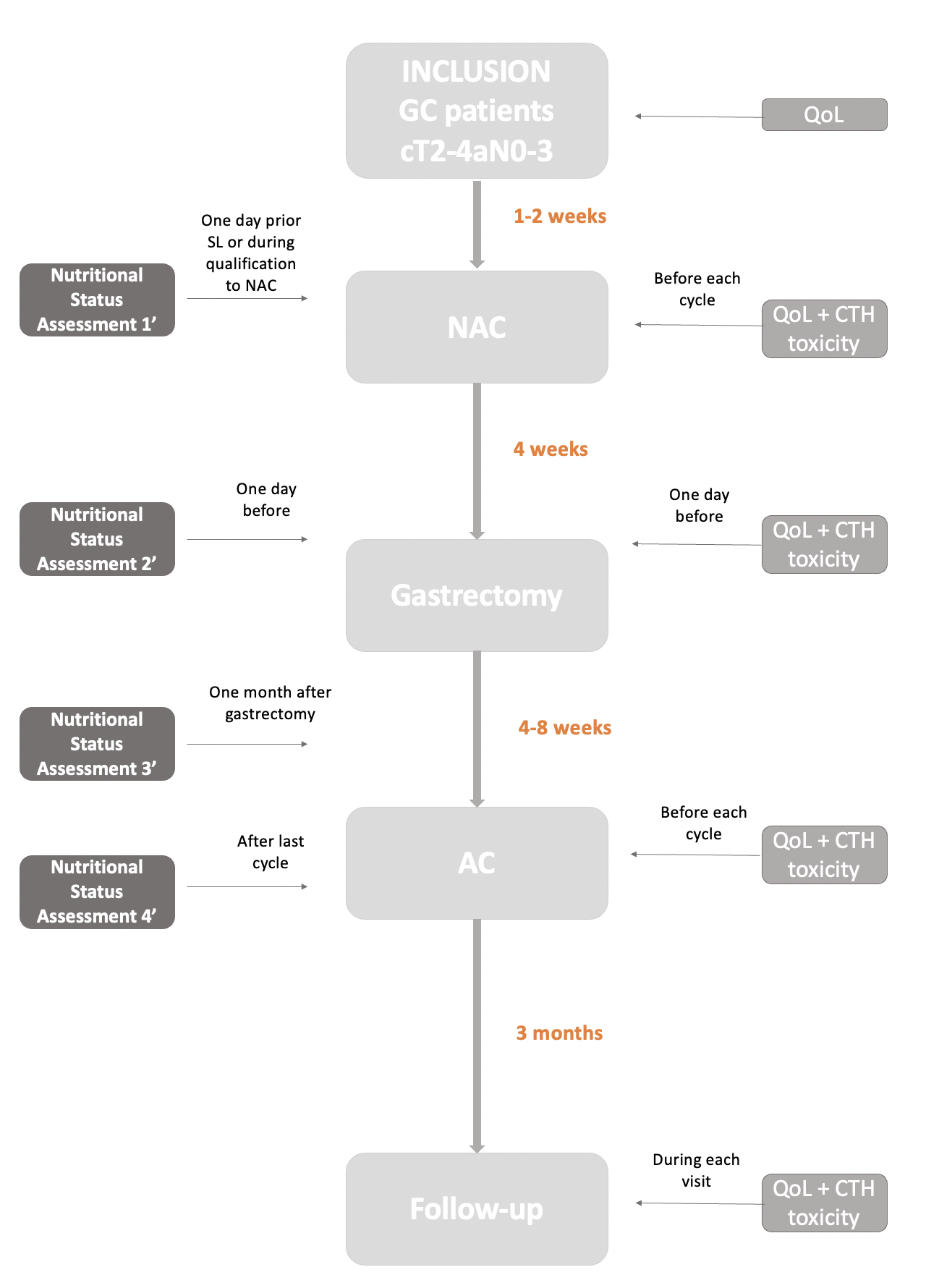


GC – gastric cancer, NAC – neoadjuvant chemotherapy, QoL – quality of life, CTH – chemotherapy, AC – adjuvant chemotherapy

**Schedule of the study**

| **Procedure/ Visit** | **Pre-screening** | **1st cycle** | **2nd cycle** | **3rd cycle** | **4th cycle** | **Surgery** | **One month after gastrctomy** | **5th cycle** | **6th cycle** | **7th cycle** | **8th cycle** | **follow-up** |
| --- | --- | --- | --- | --- | --- | --- | --- | --- | --- | --- | --- | --- |
| Patient's informed consent | X |  |  |  |  |  |  |  |  |  |  |  |
| Inclusion to the study | X |  |  |  |  |  |  |  |  |  |  |  |
| Medical history | X | X | X | X | X | X |  | X | X | X | X | X |
| Physical examination | X | X | X | X | X | X |  | X | X | X | X | X |
| Performance status | X | X | X | X | X | X |  | X | X | X | X | X |
| QoL | X | X | X | X | X | X |  | X | X | X | X | X |
| BIA | X |  |  |  | X |  | X |  |  |  | X |  |
| Dynamometry | X |  |  |  | X |  | X |  |  |  | X |  |
| Nutritional questionaries (NRS, SGA, SFDQ, NRI) | X | X | X | X | X | X | X | X | X | X | X | X |
| Laboratory tests (morphology, biochemistry, immunochemistry...) | X | X | X | X | X | X | X | X | X | X | X | X |
| Additional laboratory tests (pregnancy test, iron, ferritin, transferrin level) | X |  |  |  |  | X |  |  |  |  |  |  |
| CT scan | X |  |  |  | X* |  |  |  |  |  |  | X |
| CTH |  | X | X | X | X |  |  | X | X | X | X | X |
| CTH toxicity |  | X | X | X | X |  |  | X | X | X | X | X |

QoL – quality of life, BIA – bioimpedance analysis, NRS – nutrition risk screening, SGA - Subjective Global Assessment, SFDQ - Short Form Dietary Questionnaire, NRI – nutritional risk index, CT – computed tomography, CTH – chemotherapy, * - non-obligatory, according to the decision of the oncologist

**Aims of the study**

Based on the assessment of the endpoints:

- Overall survival (time from the beginning of systemic treatment until death)
- Tumor regression grade (TRG)
- Disease free survival (time from the initiation of systemic treatment to the first recurrence of the disease)
- Surgical radicality (percentage of R0 resections)
- Postoperative complications according to Clavien-Dindo classification and Comprehensive Cancer Index (CCI)
- Perioperative mortality (within 30 and 90 days after the surgery)
- Percentage of patients who completed treatment according to the protocol
- Toxicity of systemic treatment
- Quality of life (EORTC QLQ – STO22 questionnaire)

**Questionnaires used in the study**

All questionnaires are validated, free of charge and open access.

1. **ECOG - WHO Performance Status Scale**

A performance scale measuring the general condition and quality of life of a cancer patient.

1. [**EORTC QLQ – STO22**](https://www.eortc.org/app/uploads/sites/2/2018/08/Specimen-STO22-English.pdf)

Created by European Organization for Research and Treatment of Cancer (EORTC). This scale has been adapted by the EORTC in more than 80 language versions, including Polish.

1. **Subjective Global Assessment, SGA**

The questionnaire is an in-depth assessment of nutritional status. It consists of three parts. The first concerns general history, i.e., the patient's sex, age, height, and weight changes. This section also includes questions related to changes in food intake, type of diet, gastrointestinal symptoms, and physical performance. The second part of the questionnaire concerns the physical examination. In the third part, a subjective assessment of nutritional status is made. The SGA questionnaire provides greater insight into the patient's nutritional problems.

1. **Short Form Dietary Questionnaire, SFDQ**

A questionnaire assessing the patient's food preferences in a 'typical week' over the past month. The person completing the questionnaire indicates how often he or she eats a particular food group, with the option to provide only one answer.

1. **Nutritional Risk Score, NRS**

The questionnaire consists of two parts dedicated to adult patients. The first part contains four questions to be answered only with YES or NO. If there is at least one positive answer, proceeding to the next part of the questionnaire is necessary. However, if all answers are negative, this step should be repeated after seven days. The second part of the questionnaire assesses the disease's severity and evaluates its energy intake. If the examined patient is 70 years or older, an additional 1 point should be added to the final total.

**Inclusion criteria**

Patients with primary, locally advanced stage IIA-IIIC GC who do not require immediate surgical intervention due to underlying disease (e.g., perforation or bleeding) and no features indicative of distant metastases on imaging (CT) and staging laparoscopy will be eligible for the study.

All patients will be asked about their willingness to participate in the study to exclude selection bias. All patients will be informed of the purpose of the study and the absence of deviation from standard diagnostic and therapeutic management. Patients potentially interested in the study will be screened according to the inclusion and exclusion criteria and enrolled in the study and will be followed up to assess survival outcomes.

**BIA evaluation**

The BIA is a non-invasive and painless assessment and will not affect the treatment schedule, apart from the four electrical bioimpedance measurements (each lasting approximately 10-15 min) in the following stages of oncology treatment:

1. One day prior SL or during qualification to NAC (maximum two weeks before the start of treatment)

2. One day before the gastrectomy

3. One month after the gastrectomy

4. After the last cycle of AC

The patient undergoing the analysis stays supine for approximately 10 minutes. A total of four disposable electrodes are connected to the skin of the hands and feet. These send out pulses of current at a low intensity (0.8-1 mA) that is not perceptible to the body. The measuring device sends the data to a dedicated computer program, making appropriate calculations.

**Sample size**

The minimal study group was estimated as 125 patients.

Sample size calculation was performed based on retrospective data from the Department of Surgical Oncology of the Medical University of Lublin on patients with locally advanced GC undergoing multimodal treatment and diagnosed with malnutrition. The minimum study group was estimated at 125 patients.

**Declaration**

The study will follow the protocol and the moral, ethical, and scientific principles governing clinical trials in accordance with the Declaration of Helsinki and Good Clinical Practice (GCP). Also, the study will be conducted in accordance with local regulatory requirements and following the privacy policy in force since 25 May 2018 under Regulation (EU) 2016/679 of the European Parliament.

**Contact details:**

| **Research Theme Leader:** | **Zuzanna Pelc, M.D., PhD**  Department of Surgical Oncology, Medical University of Lublin, Radziwiłłowska 13 St., 20-080, Lublin, Poland  Phone number: +48 81-531-81-26 e-mail: [zuzanna.pelc@umlub.pl](mailto:zuzanna.pelc@umlub.pl) |
| --- | --- |
| **Head of the Department:** | **Prof. dr hab. med. Wojciech Polkowski**  Department of Surgical Oncology, Medical University of Lublin, Radziwiłłowska 13 St., 20-080, Lublin, Poland  Phone number: +48 81-531-81-26 e-mail: [wojciech.polkowski@umlub.pl](mailto:wojciech.polkowski@umlub.pl) |

Contact details of cooperating centres:

1. Upper Gastrointestinal Department of Surgery, Erasmus University Medical Centre, Rotterdam, Holandia

| **Principal Investigator:** | **Dr Pieter van der Sluis**  Upper Gastrointestinal Department of Surgery, Erasmus MC, Rotterdam, The Netherlands.  Phone number: +31 10 704 0704 e-mail: [p.vandersluis@erasmusmc.nl](mailto:p.vandersluis@erasmusmc.nl) |
| --- | --- |
| **Head of the Department:** | **Prof. Cornelis Verhoef**  Upper Gastrointestinal Department of Surgery, Erasmus MC, Rotterdam, The Netherlands  Phone number: +31 10 704 0704  e-mail: [c.verhoef@erasmusmc.nl](mailto:c.verhoef@erasmusmc.nl) |

1. Department of General, Endocrynological Surgery and Gastrointestinal Oncology, Institute of Surgery, Poznan University of Medical Sciences

| **Head of the Department and Principal Investigator:** | **Prof. dr hab. med. Tomasz Banasiewicz**  Ul. Przybyszewskiego 49, Poznań  Phone number: +48 61 869 1275 e-mail: [tbanasiewicz@op.pl](mailto:tbanasiewicz@op.pl) |
| --- | --- |

1. 2nd Department of Surgery, Jagiellonian University Medical College

| **Head of the Department and Principal Investigator:** | **Prof. dr hab. med. Michał Pędziwiatr**  Ul. Kopernika 21, 31-501 Kraków  Phone number: +48 12 400 26 30 e-mail: [mpedziwiatr@gmail.com](mailto:mpedziwiatr@gmail.com) |
| --- | --- |

Consulting and collaborating centre:

Body Composition Research Laboratory, Department of Human Physiology, Medical University of Lublin

| **Principal Investigator:** | **Dr hab. n. med. Radosław Mlak, Profesor uczelni**  Ul. Radziwiłłowska 11  Phone number: 81448 6080 e-mail: [radoslaw.mlak@gmail.com](mailto:radoslaw.mlak@gmail.com) |
| --- | --- |
| **Head of the Department:** | **Prof. dr hab. Teresa Małecka-Massalska**  Ul. Radziwiłłowska 11  Phone number: +48 81 448 60 80 e-mail: [teresa.malecka-massalska@umlub.pl](mailto:teresa.malecka-massalska@umlub.pl) |
