# Supplementary material for: MalnutritiOn assessment with biOelectrical impedaNce analysis in gastRic cancer patIentS undergoing multimodaltrEatment (MOONRISE)—Study protocol for a single-arm multicenter cross-sectional longitudinal study
Source: PLoS One. 2024 Feb 6;19(2):e0297583. doi: 10.1371/journal.pone.0297583 (PMC10846730; doi:10.1371/journal.pone.0297583)
Supplement: S2 File — (DOCX) [file pone.0297583.s002.docx]

**PROTOKÓŁ BADANIA**

**TYTUŁ BADANIA:**

**Prospektywne, wieloośrodkowe badanie oceniające niedożywienie u chorych na raka żołądka leczonych metodą skojarzoną za pomocą bioimpedancji elektrycznej (Badanie MOONRISE).**

**KIEROWNIK TEMATU BADAWCZEGO:**

Lek. Zuzanna Pelc

**OŚRODEK:**

Uniwersytet Medyczny w Lublinie:

Klinika Chirurgii Onkologicznej UM w Lublinie

**DOTYCZĄCE BADANIA NUMERY TELEFONÓW:**

Klinika Chirurgii Onkologicznej UM w Lublinie:

81-531-81-26

Rak żołądka (RŻ) pozostaje jednym z najczęściej diagnozowanych nowotworów złośliwych oraz czwartą główną przyczyną zgonów onkologicznych na świecie. Zastosowanie chemioterapii okołooperacyjnej oraz stały postęp w jakości chirurgii poprawiły wyniki leczenia, jednak istnieje dalsza potrzeba indywidualizacji terapii w celu zmniejszenia zachorowalności i śmiertelności chorych onkologicznych.

Mimo wyraźnych postępów w zrozumieniu biologii RŻ, leczenie operacyjne pozostaje standardem postępowania w chorobie miejscowo zaawansowanej. Wprowadzenie do praktyki klinicznej leczenia skojarzonego, poprzedzającego i uzupełniającego leczenie operacyjne chemioterapią, wymusza weryfikację optymalnego zakresu wycięcia zarówno ogniska pierwotnego jak i regionalnych węzłów chłonnych.

Zastosowanie chemioterapii neoadjuwantowej wydaje się zarówno zwiększać radykalność resekcji, jak i eliminować wczesny rozsiew choroby, jednocześnie pozwalając na kliniczną i histopatologiczną ocenę odpowiedzi na leczenie.

Chociaż leczenie przedoperacyjne zwiększa odsetek resekcyjności ogniska pierwotnego, jego toksyczność obniża jakość życia pacjentów. Ponadto, u około 30% chorych na RŻ po leczeniu operacyjnym występuje niezamierzona utrata masy ciała, a u chorych po 65 roku życia, wskaźnik niedożywienia sięga nawet 70%.

Niedożywienie przyczynia się do zwiększenia zachorowalności, wzrostu odsetka powikłań okołooperacyjnych i nasilonej toksyczności ogólnoustrojowej. Skutkuje wydłużeniem pobytu w szpitalu, obniżeniem jakości życia, zmniejszeniem przeżywalności. Zależność między stanem odżywienia a przeżyciem całkowitym u chorych na miejscowo-zaawansowanego RŻ leczonych metodą skojarzoną jest niejednoznaczna. Niezależnymi czynnikami rokowniczymi jest natomiast utrata masy ciała, zarówno przed operacją, jak i po zakończonym leczeniu chirurgicznym. Ponadto, niedożywienie sprzyja postępowi i agresywnemu przebiegowi choroby, a związek z przewlekłym stanem zapalnym implikuje upośledzenie układu immunologicznego, prowadząc do zwiększonego ryzyka infekcji pooperacyjnych i przerzutów odległych.

Jednym z nieinwazyjnych i obiektywnych sposobów śledzenia zmian w składzie ciała jest analiza bioimpedancji elektrycznej (ang. bioelectrical impedance analysis, BIA). Badanie BIA polega na pomiarze impedancji (czyli rodzaju oporu elektrycznego złożonego z rezystancji i reaktancji tkanek, przez które przepuszczany jest prąd o niskim natężeniu - 0.8-1mA). Rezystancja wiąże się z oporem właściwym poszczególnych tkanek, podczas gdy reaktancja wynika głównie z pojemności elektrycznej i budowy błon komórkowych. BIA może ujawnić pierwotne objawy niedożywienia nawet na kilka miesięcy przed kacheksją, a brak ekspozycji na promieniowanie i niski koszt testu pozwala na regularne śledzenie zmian w składzie ciała.

# Projekt badania

Jest to prospektywne, wieloośrodkowe badanie obserwacyjne, którego celem jest ocena niedożywienia chorych na zaawansowanego RŻ leczonych metodą skojarzoną przy pomocy BIA.

## Schemat badania


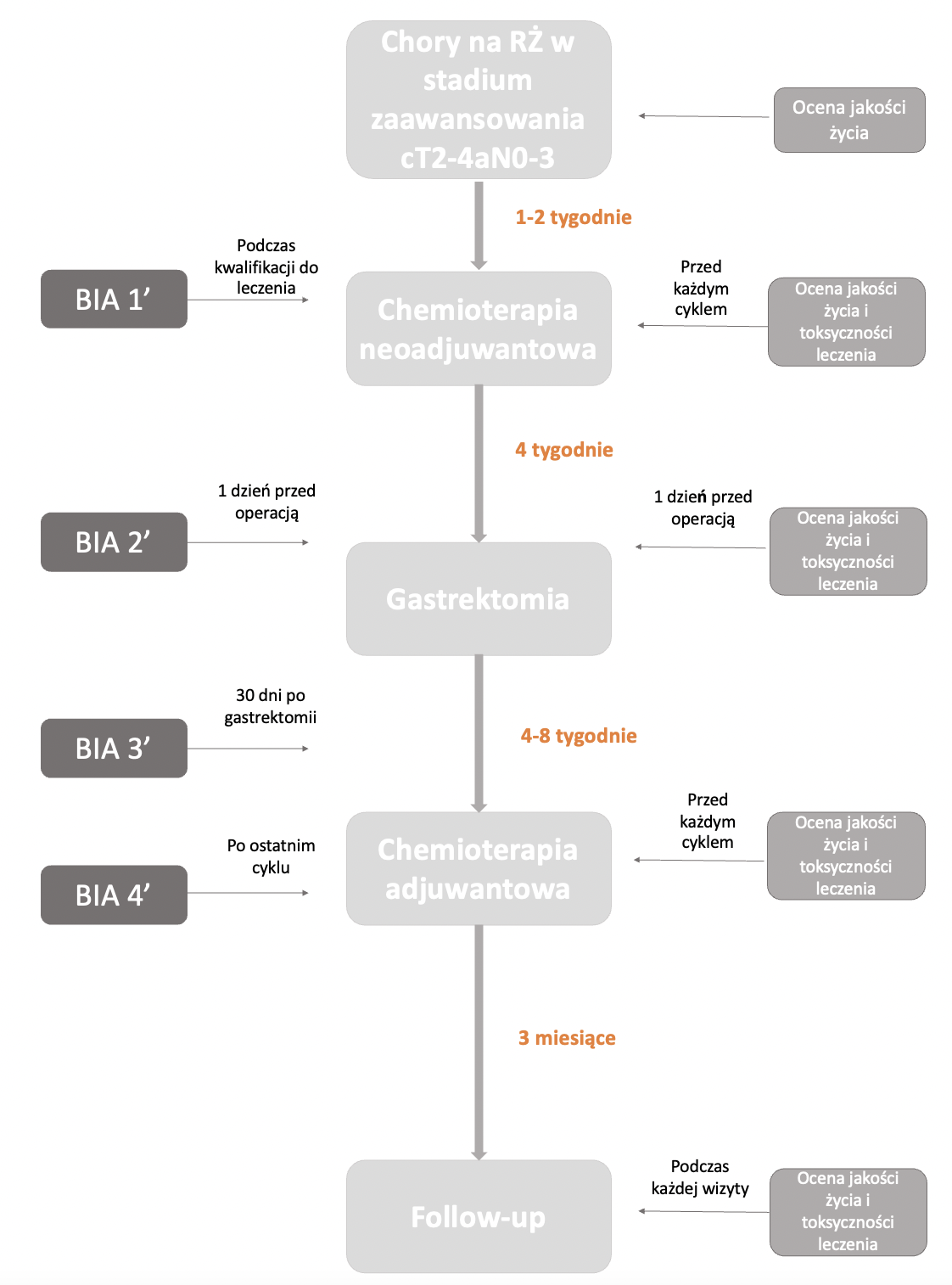


BIA – bioimpedancja elektryczna, RŻ – rak żołądka

**Przebieg badania**

BIA – bioimpedancja elektryczna, NRS – Ocena Ryzyka Związanego Ze Stanem Odżywienia, SGA - Subiektywna Globalna Ocena Stanu Odżywienia, SFDQ – Skrócony kwestionariusz oceny sposobu żywienia, * - cykl chemioterapii przedoperacyjnej, ** - cykl chemioterapii pooperacyjnej, ^ - niewymagana

| **Procedura / Wizyta** | **Wizyta pilotażowa** | **I* cykl** | **II cykl** | **III cykl** | **IV cykl** | **Operacja** | **V** cykl** | **VI cykl** | **VII cykl** | **VIII cykl** | **Follow-up** |
| --- | --- | --- | --- | --- | --- | --- | --- | --- | --- | --- | --- |
| Pisemna zgoda chorego | X |  |  |  |  |  |  |  |  |  |  |
| Włączenie chorego do badania | X |  |  |  |  |  |  |  |  |  |  |
| Wywiad | X | X | X | X | X | X | X | X | X | X | X |
| Badanie fizykalne | X | X | X | X | X | X | X | X | X | X | X |
| Ocena sprawności | X | X | X | X | X | X | X | X | X | X | X |
| Ocena jakości życia | X | X | X | X | X | X | X | X | X | X | X |
| BIA | X |  |  |  | X | X |  |  |  | X |  |
| Dynamometria | X |  |  |  | X | X |  |  |  | X |  |
| Ocena st. odżywienia (ankiety NRS, SGA, SFDQ) | X | X | X | X | X | X | X | X | X | X | X |
| Badania laboratoryjne | X | X | X | X | X | X | X | X | X | X | X |
| Dodatkowe badania laboratoryjne (β-HCG, żelazo, ferrytyna, transferryna) | X |  |  |  |  | X |  |  |  |  |  |
| Tomografia komputerowa | X |  |  |  | X^ |  |  |  |  |  | X |
| Chemioterapia |  | X | X | X | X |  | X | X | X | X |  |
| Ocena toksyczności chemioterapii |  | X | X | X | X |  | X | X | X | X | X |

**Cele badania**

Opierają się na ocenie punktów końcowych:

- Przeżycie całkowite (jako czas od rozpoczęcia leczenia systemowego do śmierci)
- Ocena stopnia odpowiedzi leczenia systemowego (ang. tumour regression grade, TRG) na ognisko pierwotne
- Czas przeżycia bez nawrotu (jako czas od rozpoczęcia leczenia systemowego do pierwszego nawrotu choroby)
- Radykalność chirurgiczna (odsetek resekcji R0)
- Powikłania pooperacyjne według klasyfikacji Clavien-Dindo oraz Comprehensive Cancer Index (CCI)
- Śmiertelność okołooperacyjna (w ciągu 30 i 90 dni od operacji)
- Procent chorych, którzy ukończyli leczenie zgodnie z protokołem
- Toksyczność chemioterapii
- Jakość życia chorego (kwestionariusz EORTC QLQ – STO22)

**Ankiety stosowane w trakcie badania**

Wszystkie kwestionariusze są walidowane, bezpłatne i powszechnie dostępne.

1. **Skala Zubroda-ECOG-WHO**

Skala sprawności pozwalająca określić stan ogólny i jakość życia pacjenta z chorobą nowotworową.

1. **Kwestionariusz oceny jakości życia** [**EORTC QLQ – STO22**](https://www.eortc.org/app/uploads/sites/2/2018/08/Specimen-STO22-English.pdf)

Został utworzony przez European Organization for Research and Treatment of Cancer (EORTC). Skala ta została zaadaptowana przez EORTC w ponad 80 wersjach językowych, w tym również w polskiej.

1. **Skala Subiektywnej Całościowej Oceny Stanu Odżywienia (ang. Subjective Global Assessment, SGA)**

Kwestionariusz stanowi pogłębioną ocenę stanu odżywienia. Składa się z trzech części. Pierwsza dotyczy wywiadu ogólnego tj. płci, wieku, wzrostu i zmian masy ciała chorego. W tej części znajdują się również pytania związane ze zmianami przyjmowania pokarmów, rodzajem diety, objawami ze strony przewodu pokarmowego oraz wydolnością fizyczną. Druga część kwestionariusza dotyczy badania fizykalnego. W części trzeciej dokonuje się subiektywnej oceny stanu odżywienia.. Kwestionariusz SGA zapewnia głębszą możliwość poznania problemów żywieniowych chorego.

1. **Skrócony kwestionariusz oceny sposobu żywienia (ang. Short Form Dietary Questionnaire, SFDQ)**

Kwestionariusz oceniający preferencje żywieniowe chorego w „typowym tygodniu“ w ciągu ostatniego miesiąca. Wypełniający zaznacza jak często spożywa daną grupę pokarmów, mając możliwość udzielenia tylko jednej odpowiedzi.

1. **Skala Oceny Ryzyka Żywieniowego 2002 (ang. Nutritional Risk Score, NRS)**

Kwestionariusz składa się z dwóch części dedykowany dorosłym chorym. Pierwsza część zawiera 4 pytania, na które należy odpowiedzieć jedynie TAK lub NIE. W przypadku uzyskania przynajmniej jednej odpowiedzi twierdzącej, należy przejść do kolejnej części kwestionariusza. Jeżeli jednak wszystkie odpowiedzi są zaprzeczające, należy powtórzyć ten etap po 7 dniach. Druga część kwestionariusza ocenia nasilenie choroby oraz ocenia zapotrzebowanie energetyczne jakie z niej wynika Jeżeli wiek chorego, którego oceniamy tym formularzem przekracza 70 lat, należy doliczyć dodatkowy 1 punkt, do końcowej sumy.

**Kryteria doboru pacjentów**

Do badania kwalifikowani będą chorzy na pierwotnego, miejscowo-zaawansowanego raka żołądka w stadium IIA-IIIC, którzy nie wymagają natychmiastowej interwencji chirurgicznej z powodu choroby podstawowej (np. perforacja lub krwawienie) i brakiem cech wskazujących na obecność przerzutów odległych w badaniach obrazowych (TK) oraz laparoskopii diagnostycznej.

Wszyscy chorzy zostaną zapytani o chęć udziału w badaniu celem wykluczenia błędów selekcji. Wszyscy chorzy zostaną poinformowani o celu badania i braku odstępstw od standardowego postępowania diagnostyczno-terapeutycznego. Chorzy potencjalnie zainteresowani badaniem zostaną poddani badaniom skriningowym zgodnie z kryteriami włączenia i wykluczenia oraz zarejestrowani do badania, jak również zostaną poddani obserwacji (follow-up) celem oceny wyniku przeżycia.

**Ocena BIA**

Badanie bioimpedancji elektrycznej jest nieinwazyjne i bezbolesne, oraz nie będzie miało wpływu na sposób przeprowadzenia schematu leczenia skojarzonego chorych na miejscowo zaawansowanego RŻ, poza koniecznością przeprowadzenia 4 pomiarów bioimpedancji elektrycznej (każdy trwający ok 10-15 min.) w następujących etapach leczenia onkologicznego:

1. Podczas kwalifikacji do chemioterapii neoadjuwantowej (maksymalnie 2 tygodnie przez rozpoczęciem leczenia)

2. Dzień przed leczeniem operacyjnym

3. Miesiąc po leczeniu operacyjnym

4. Po zakończonej chemioterapii adjuwantowej

Chory poddany analizie przebywa przez ok. 10 min. w pozycji leżącej. Do skóry dłoni i stóp zostają podłączone łącznie 4 jednorazowe elektrody. Wysyłają one impulsy prądu o niskim, niewyczuwalnym dla organizmu natężeniu (0,8-1 mA). Urządzenie mierzące przesyła dane do dedykowanego programu komputerowego, który dokonuje właściwych obliczeń.

**Liczba uczestników**

Ogólna wielkość próby: 125 chorych

Obliczenie wielkości próby zostało wykonane na podstawie danych retrospektywnych pochodzących z Kliniki Chirurgii Onkologicznej UM w Lublinie dotyczących chorych na lokalnie zaawansowanego RŻ poddanych leczeniu skojarzonemu u których zdiagnozowano niedożywienie. Minimalną grupę badaną oszacowano na 125 chorych.

**Oświadczenie**

Badanie zostanie przeprowadzone zgodnie z protokołem i z zasadami moralnymi, etycznymi i naukowymi regulującymi badania kliniczne, zgodnie z Deklaracją Helsińską i Dobrą Praktyką Kliniczną (GCP). Badanie zostanie również przeprowadzone zgodnie z lokalnymi i regulacyjnymi wymogami, zgodnie z polityką prywatności obowiązującą od 25 maja 2018 r. na podstawie Rozporządzenia (UE) 2016/679 Parlamentu Europejskiego.

**Dane kontaktowe:**

| **Kierownik Tematu Badawczego:** | **Lek. Zuzanna Pelc**  Klinika Chirurgii Onkologicznej UM w Lublinie  Ul. Radziwiłłowska 13  Telefon: 81-531-81-26 e-mail: [zuzanna.pelc@umlub.pl](mailto:zuzanna.pelc@umlub.pl) |
| --- | --- |
| **Kierownik Kliniki:** | **Prof. dr hab. med. Wojciech Polkowski**  Klinika Chirurgii Onkologicznej UM w Lublinie  Ul. Radziwiłłowska 13  Telefon: 81-531-81-26 e-mail: [wojciech.polkowski@umlub.pl](mailto:wojciech.polkowski@umlub.pl) |

Dane kontaktowe ośrodków współpracujących

1. Klinika Chirurgii, Erasmus University Medical Centre, Rotterdam, Holandia

| **Badacz Główny:** | **Dr Pieter van der Sluis**  Klinika Chirurgii, Erasmus University Medical Centre  Doctor Molewaterplein 40  Telefon: +31 10 704 0704 e-mail: [p.vandersluis@erasmusmc.nl](mailto:p.vandersluis@erasmusmc.nl) |
| --- | --- |
| **Kierownik Kliniki:** | **Prof. Cornelis Verhoef**  Klinika Chirurgii, Erasmus University Medical Centre  Doctor Molewaterplein 40  Telefon: +31 10 704 0704  e-mail: [c.verhoef@erasmusmc.nl](mailto:c.verhoef@erasmusmc.nl) |

1. Katedra i Klinika Chirurgii Chirurgii Ogólnej, Endokrynologicznej i Onkologii Gastroenterologicznej Uniwersytetu Medycznego im. Karola Marcinkowskiego w Poznaniu

| **Kierownik Kliniki i Badacz Główny:** | **Prof. dr hab. med. Tomasz Banasiewicz**  Ul. Przybyszewskiego 49, Poznań  Telefon: 61 869 1275 e-mail: [tbanasiewicz@op.pl](mailto:tbanasiewicz@op.pl) |
| --- | --- |

1. II Katedra Chirurgii Ogólnej, Uniwersytet Jagielloński Collegium Medicum w Krakowie

| **Kierownik Kliniki i Badacz Główny:** | **Prof. dr hab. med. Michał Pędziwiatr**  Ul. Kopernika 21, 31-501 Kraków  Telefon: 12 400 26 30 e-mail: [mpedziwiatr@gmail.com](mailto:mpedziwiatr@gmail.com) |
| --- | --- |

Ośrodek konsultujący i współpracujący:

Pracownia Badań Składu Ciała Katedry i Zakładu Fizjologii Człowieka Uniwersytetu Medycznego w Lublinie

| **Kierownik Pracowni i Badacz Główny:** | **Dr hab. n. med. Radosław Mlak, Profesor uczelni**  Ul. Radziwiłłowska 11  Telefon: 81448 6080 e-mail: [radoslaw.mlak@gmail.com](mailto:radoslaw.mlak@gmail.com) |
| --- | --- |
| **Kierownik Katedry i Zakładu:** | **Prof. dr hab. Teresa Małecka-Massalska**  Ul. Radziwiłłowska 11  Telefon: 81448 6080 e-mail: [teresa.malecka-massalska@umlub.pl](mailto:teresa.malecka-massalska@umlub.pl) |
